# The ‘Neglected’ Personal Neglect

**DOI:** 10.1007/s11065-018-9394-4

**Published:** 2018-12-13

**Authors:** Pietro Caggiano, Mervi Jehkonen

**Affiliations:** 1 Psychology Department, Goldsmiths University of London, New Cross, London, SE14 6NW UK; 2 Faculty of Social Sciences, University of Tampere, Tampere, Finland; 3 Department of Neurology and Rehabilitation, Tampere University Hospital, Tampere, Finland

**Keywords:** Anosognosia, Assessment, Brain damage, Hemispatial neglect, Neuropsychology, Personal neglect, Extrapersonal neglect, Stroke

## Abstract

A review of patients with brain injury showing personal neglect is presented. The aim is to shed light on this aspect of neglect often unresearched or only indirectly investigated, and to discuss recent findings concerning the methods used to assess personal neglect, its neural correlates and its association with the more often explored aspect of extrapersonal neglect. The review was performed using PubMed and PsychInfo databases to search for papers published in the last 123 years (until January 2018). We reviewed 81 papers describing either single or group studies for a total of 2247 patients. The results of this review showed that various aspects of personal neglect are still controversial and outcomes potentially contradictory. Despite the data reported in the present review suggest that personal neglect is more frequently associated with lesions of the right hemisphere, the left hemisphere may also play an important role. Not surprisingly, personal neglect and extrapersonal neglect seem to co-occur. However double dissociations of these two forms of neglect have been reported, and they seem to dissociate both from a functional and an anatomical perspective. More recent interpretations of personal neglect suggest that it may result from a disrupted body representation. The development of reliable psychometric tools with shared diagnostic criteria is essential to identify different degrees of personal neglect for different body parts and to better refine personal neglect in comparison to extrapersonal neglect and disorders related to distortions of personal domain.

## Introduction

Hemispatial neglect is a well-known and relatively common deficit following unilateral brain lesions and it refers to a variety of acquired neuropsychological disorders that affect spatial cognition (see Halligan, Fink, Marshall, & Vallar, 2003; Vallar, 1998 for reviews). The main feature of hemispatial neglect is a general lack of awareness and attention to stimuli located in the contralesional side of space and not due to elementary sensory or motor disorders. Hemispatial neglect can be fractionated into different patterns of impairment according to the specific frame of reference (personal, reaching space and far extrapersonal) that can be selectively affected according to distinct coordinates (Vallar, 1998).

Personal neglect refers to a form of hemi-inattention where brain-injured patients show a “deficit relative to the side of the body contralateral to the lesion” (Guariglia & Antonucci, 1992; p.1001). This definition of ‘personal neglect’ seems to imply a general inattention for the contralesional side of the body. However, different attentional deficits can be related to the contralesional limb. In 1893, Gabriel Anton described the case of a patient who exhibited a deficit of tactile and pain sensation on the left side, as well as proprioceptive impairment of position sense. The loss of both the perception and the “knowledge” of the left side fits with the current definition of personal neglect. However, it is widely accepted in the neuropsychological literature that the first clear observation of personal neglect was made in 1913, by Hermann Zingerle, who referred to Anton’s case. Zingerle described two patients with right brain-injury (cases 2 and 3) both of whom failed to spontaneously attend to the left side of their own body. Referring to case 2, Zingerle noted that the patient “never drew his attention to it [ …] apparently never missed it, and seemed to have completely forgotten about it” (translation as in Benke, Luzzatti, & Vallar, 2004; p. 268). With case 3, Zingerle stated that “the patient did not use his left arm at all for spontaneous movements [ …] it was impossible to obtain comments about his left side or a description of the sensations on that side.” (translation as in Benke et al., 2004; p. 270). Zingerle’s early descriptions seem to reflect different possible deficits associated with neglect in the personal domain: motor and premotor neglect. The term motor neglect (Laplane & Degos, 1983) refers to patients that show a considerable reduction in spontaneous use of their contralesional limbs (as in Zingerle’s case 3), which is not explained by their associated motor impairments (e.g., Punt & Riddoch, 2006; Sampanis & Riddoch, 2013). On the other hand, premotor neglect (Heilman, Bowers, Coslett, Whelan, & Watson, 1985; Bisiach, Vallar, Perani, Papagno, & Berti, 1986b) refers to patients, like Zingerle’s cases 2 and 3, who show a reduced tendency to perform movements with the ipsilesional (unimpaired) limbs toward the contralesional side of their body (Bisiach, Perani, Vallar, & Berti, 1986a) as well as within peripersonal space (Saevarsson, Eger, & Gutierrez-Herrera, 2014).

The relationship between motor and premotor neglect is still unclear and investigation of these two forms of neglect is rarely carried out in the same sample (e.g., Garbarini et al., 2012; Buxbaum et al., 2004). Despite the fact that both motor and premotor neglect within personal space should intuitively fall under the umbrella of ‘personal neglect’, the term personal neglect has often been used to identify the premotor form. As such, we re-direct the reader interested in motor neglect to a review by Punt and Riddoch (2006; see also Sampanis & Riddoch, 2013), as this review will mainly focus on personal neglect defined as a set of spatially asymmetrical symptoms occurring in personal space including: defective awareness of the contralesional side of the body, a lack of awareness of contralesional tactile or proprioceptive stimuli and defective motor programming towards targets in the neglected sector of space (Vallar, 1998). Patients tend to neglect the contralesional side of their body, for example, they may forget to shave the left side of their face or fail to properly dress the left side of their body (e.g. not put the left arm in the left sleeve of a shirt).

The aim of this review is to elucidate different aspects and findings of this specific type of neglect, which is often overlooked or investigated indirectly, by appraising research undertaken since Anton’s first description.

## Studies on Personal Neglect: A Systematic Literature Review

The literature on personal neglect is quite fragmented, with inconsistent definitions and assessment methods. This lack of clarity causes difficulties in understanding and defining such an impairment and has implications for both theoretical and clinical or diagnostic purposes. A recent review from Committeri, Piervincenzi and Pizzamiglio (2018) provides a useful re-evaluation of the theoretical accounts and neuro-anatomical correlates of personal neglect, highlighting the association of personal neglect with a variety of body representation disorders. The present review offers a systematic update on relevant research and frames personal neglect in terms of its assessment methods, neural substrates and its relationship with inattention for extrapersonal space.

## Method

The review was performed using PubMed and PsychInfo databases to search for papers published before January 2018. Following an initial search with the keywords: *personal neglect and stroke*; *personal neglect and brain damage*; *personal neglect and lesion; body representation neglect,* we identified 62 papers that mentioned personal neglect as part of an empirical study or review paper.

We initially considered English language papers only, though crucial, historical papers in different languages (e.g. Anton, 1893; Zingerle, 1913) were also considered. As part of a second additional search, we scrutinized various papers and reviews on neglect (i.e., Azouvi et al., 2006; List, Brooks, Esterman, & Flevaris, 2008; Rode, Pagliari, Huchon, Rossetti, & Pisella, 2016; Vallar & Ronchi, 2009; Jehkonen, Laihosalo, & Kettunen, 2006) and on associated disorders, such as anosognosia (e.g., Appelros, Karlsson, Seiger, & Nydevik, 2002; Cocchini, Beschin, & Sala, 2002; Buxbaum et al., 2004; Berti, Làdavas, & Della Corte, 1996) to identify further studies about personal neglect, some of which were not published in English. Additionally, for the purpose of the present review, we did not consider forms of neglect which explicitly referred to the clinical description of motor neglect provided by Laplane and Degos (1983) and schematized in the work of Punt and Riddoch (2006).

## Results and Discussion

We finally considered a sample of 81 papers (51 reporting single cases and 30 reporting group studies) published in the last 123 years (between 1893 and 2018). The number of patients in the 81 studies ranged from 1 (single case studies) to 282 (group studies). Table 1 reports demographic and clinical information of 51 papers describing 83 single or multiple cases showing personal neglect. Table 2 includes 30 group studies in which personal neglect was assessed as the primary purpose of the studies or as a control variable. The sample size of group studies ranges from eight (Reinhart et al., 2012) to 282 (Guariglia et al., 2014) patients for a total sample size of 2247 patients, of which 755 were reported to have personal neglect (33.6%). Details of these studies are discussed in the sections below considering three aspects that we consider crucial in order to better understand the implications of personal neglect for future research and clinical purposes i) assessment methods, ii) neural correlates of personal neglect and iii) the relationship of personal neglect with extrapersonal neglect.

**Table 1:** Demographic, clinical and neuropsychological data of 83 brain-injured patients showing evidence of personal neglect described in 51 single-case studies

Author/s	Assessment method	Case N	Gender	Age/ mean age	Aetiology	Onset (days)	Lesion side	Lesion site (main structures)	EN
Anton, 1893	Clinical observation	#1	M	65	Vascular	na	R	O, thalamus	+
Zingerle, 1913	Clinical observation	#2	M	45	Vascular	‘acute’	R	na	na
Kramer, 1915	Clinical observation	#1	M	na	Vascular	na	R	na	+
Barré, Morin, & Kaiser, 1923	Clinical observation	#1	M	60	Vascular	na	R	na	+
Barkman, 1925	Clinical observation	#9	F	53	Vascular	na	R	na	+
Ehrenwald, 1930	Clinical observation	#1	M	59	Vascular	na	R	na	na
Ehrenwald, 1931	Clinical observation	#5	M	64	Vascular	na	R	na	+
Potzl quoted by Schilder (1935)	Clinical observation	#1	M	na	Vascular	na	R	P, internal capsule	+
		#2	M	na	Vascular	na	R	P, O, thalamus	na
Lhermitte & Tchehrazi, 1937	Clinical observation	#1	M	70	Vascular	na	R	F, T, P	na
Von Hagen & Ives, 1937	Clinical observation	#1	F	48	Vascular	na	R	na	+
Garcin, Varay, & Hadji-Dimo, 1938	Clinical observation	#1	M	64	Tumor	na	R	T, P	–
Rubinstein, 1941	Clinical observation	#4	F	63	Vascular	na	R	na	+
Wortis & Dattner, 1942	Clinical observation	#1	F	78	Vascular	na	R	F,T,P (MCI territory)	+
Gerstmann, 1942	Clinical observation	#2	F	38	Vascular	na	R	na	+
Sandifer, 1946	Clinical observation	#1	F	66	Vascular	na	R	P, T, thalamus	+
Roth, 1949	Clinical observation	#1	F	61	Tumor	na	L/R	P, F, T, white matter	+
		#2	M	51	Vascular	na	R	ICA territory	na
Weinstein & Kahan, 1950	Clinical observation	#7	F	38	Tumor	na	R	T	na
Weinstein, Kahn, Malitz, & Rozanski, 1954	Clinical observation	#1	F	57	Vascular & Tumor	na	R	cingulate gyrus	+
Frederiks, 1963	Clinical observation	#1	M	36	Vascular	na	R	T	na
Verret & Lapresle, 1978	Clinical observation	#1	F	64	Vascular	na	R	T	na
Healton, Navarro, Bressman, & Brust, 1982	Clinical observation	#1	F	75	Vascular	na	R	basal ganglia, lenticular nucleus, external capsule	+
Nightingale, 1982	Clinical observation	#1	M	46	Tumor	na	R	P	na
Assal, 1983	Clinical observation	#1	F	86	Vascular	na	R	T	+
Berthier & Starkstein, 1987	Clinical observation	#1	M	63	Vascular	na	R	F T P	+
Starkstein, Berthier, Fedoro, Price, & Robinson, 1990	One Item test	#1	M	71	Vascular	na	R	F, cingulate gyrus, corpus callosum	+
		#2	F	48	Vascular	na	R	Territory of MCA	+
Bisiach, Meregalli, & Berti, 1990	One Item test	#1	M	74	Vascular	na	R	T, P, O	+
Rode et al., 1992	One Item Test	#1	F	69	Vascular	na	R	P, T, O, subcortical structures	+
Zoccolotti & Judica, 1991	SFES-P	#1	M	43	Vascular	na	L/R	right F, T, P; left cerebellum	–
Guariglia & Antonucci, 1992	SFES-P, BRT	#1	M	42	Vascular	na	R	F, P, subcortial structures	–
Halligan, 1995	Clinical observation	#1	M	41	Vascular	na	R	T, P	+
Aglioti, Smania, Manfredi, & Berlucchi, 1996	na	#1	F	73	Vascular	4	R	F,T,P,O, subcortical structures	+
Beschin, Cocchini, Della Sala, & Logie, 1997	One item test, Fluff test, Comb and Razor/ Compact	#1	M	67	Vascular	16	R	P	–
Peru & Pinna, 1997	One item test	#1	F	56	Vascular	35	L	T	–
Coslett, 1998	Clinical observation	#1	na	72	na	730	R	F, T, P	+
		#2	na	52	na	21	R	F, P	+
		#3	M	67	Vascular	365	R	P	+
Schiff & Pulver, 1999	Clinical observation	#1	F	81	Vascular	na	L	F, T, P, O	+
Paulig, Weber, & Garbelotto, 2000	Clinical observation	#1	F	85	Vascular	na	R	PCA territory, including deep branches (thalamus)	+
Beschin, Basso, & Della Sala, 2000	One item test, Fluff test, Comb and Razor/ Compact	#1	M	67	Vascular	1460	L/R	left P, O, right thalamus	+
Tei, 2000	Clinical observation	#1	F	76	Vascular	2	R	F, P	+
Bottini, Bisiach, Sterzi, & Vallar, 2002	One item test	#1	F	77	Vascular	‘acute’	R	insula, internal capsule, others white matter tracts	+
Cocchini et al., 2002	Comb and Razor/ Compact, Fluff test, One item test	#1	M	27	TBI	182	R/L	left & right F; right F, P, internal capsule; left T	+
Maguire & Ogden, 2002	One item test	#1	F	54.8	Vascular	28–42	L	F, T, thalamus, basal ganglia	+
		#2	F		Vascular		R	F, T, basal ganglia	+
		#3	F		Vascular		R	F, T, P, basal ganglia	+
		#4	M		Vascular		R	F, T, P, basal ganglia	+
		#5	M		Vascular		R	F, T, P, basal ganglia	+
		#6	M		Vascular		R	F, T, basal ganglia	+
		#7	M		Vascular		R	F, T, P, basal ganglia	+
		#8	M		Vascular		R	F, P basal ganglia	+
		#9	M		Vascular		R	F, P basal ganglia	+
Marangolo, Piccardi, & Rinaldi, 2003	SFES-P, BRT	#1	M	78	Vascular	122	R	F-P, T, subcortical structures	–
Moro, Zampini, & Aglioti, 2004	Comb and Razor/ Compact	#1	F	62	Vascular	213	R	F, T, P	+
		#2	M	66	Vascular	33	R	F,T,P, subcortical structures	+
Ortigue, Mégevand, Perren, Landis, & Blanke, 2006	One item test, Fluff test, Comb and Razor/ Compact	#1	M	67	Vascular	‘acute’	R	internal capsule	–
Garbarini et al., 2012	Fluff test	#1	na	66	Vascular	62	R	F, P, thalamus, external capsule, caudate	+
		#2	na	84	Vascular	32	R	P, putamen, internal and external capsule, insula, others	+
		#4	na	76	Vascular	27	R	F, T, P, internal and external capsule, others	+
		#6	na	62	Vascular	36	R	T, P, thalamus, posterior insula, others	+
		#8	na	79	Vascular	31	R	O, T, P, posterior insula and internal capsule	+
		#10	na	71	Vascular	30	R	F, T, others	+
Sambo et al., 2012	One Item Test, Fluff test	#1	M	77	Vascular	425	R	basal ganglia	+
	One Item Test, Fluff test	#2	M	36	Vascular	365	R	F,T basal ganglia	+
	One Item Test, One Item (extended)	#3	M	76	Vascular	335	R	F,T,P	+
	One Item Test, One Item (extended)	#4	M	69	Vascular	30	R	F,T,P	+
Invernizzi et al., 2013	One item test	#1	F	60	Vascular	5	R	thalamus, basal ganglia	+
		#2	F	69	Vascular	3	R	F, T,P, basal ganglia, thalamus	+
		#3	M	71	Vascular	5	R	thalamus, basal ganglia	+
		#4	F	84	Vascular	21	R	T, O, thalamus, basal ganglia, internal capsule	+
		#5	M	70	Vascular	1	R	thalamus, basal ganglia, internal capsule	+
Di Vita, Palermo, Piccardi, & Guariglia, 2015	SFES-P, BRT	#1	M	63	Vascular	10	R	F, T, P, insula	+
Di Vita et al., 2016	SFES-P, BRT	#2	F	75	Vascular	10–90	L	insula, external capsule, putamen	+
		#8	M	47	Vascular		R	internal capsule, corona radiata	+
		#11	M	74	Vascular		R	F, T	–
		#12	F	65	Vascular		R	na	+
		#14	M	64	Vascular		R	temporal-parietal junction	–
		#15	M	58	Vascular		R	P	–
		#18	M	74	Vascular		R	striatum, capsule	–
Facchin, Beschin, & Daini, 2016	Comb and Razor/ Compact, Fluff test	#1	M	49	Vascular	180	L	T,P, insula, putamen, internal capsule, superior lateral fasciculus	+
Ronchi, Heydrich, Serino, & Blanke, 2017	One Item test, Fluff test	#1	M	58	Vascular	14	R	subcortical	+
Total number of patients		83	M = 46; F = 29; na = 8	Mean = 63.2 SD = 13.4	Vascular = 75; Tumor = 4; Vascular&Tumor = 1; TBI = 1; na = 2	Mean = 152.5 SD = 290.9#	76 RBD; 5 LBD; 2 Bilateral	Total number of patients showing associated disorders	63/74 (85.1%)

*SFES-P*, Semistructured Functional Evaluation Scale - Personal sub-scale; *BRT*, Body Representation Test; *TBI*, Traumatic Brain Injury; *LBD*, Left Brain Damage; *RBD*, Right Brain Damage; *F*, Female; *M*, Male; *na*, not available; *F*, Frontal, *T*, Temporal; *P*, Parietal; *O*, Occipital lobes; *R*, Right; *L*, Left; *MCA*, Middle Cerebral Artery; *ICA* Inferior Cerebral Artery; *PCA*, Posterior Cerebral Artery; *PN*, Personal Neglect; *EN*, Extrapersonal Neglect; ‘*acute*’, as reported by authors; less than 7 days; *na*, information not available

+ deficit present

− deficit absent

#where range was available, the average of the group onset was considered

**Table 2:** Assessment method, clinical and neuropsychological details of the 30 group studies reporting evidence of personal neglect

Author/s	Assessment method	Aetiology	Lesion side	Lesion site (main structures)	PN (sample)	PN %	EN (PN)	
Cutting, 1978	Clinical observation	Vascular	R	na	10 (48)	21	10 (10)	
Bisiach et al., 1986a, b	One item test	Vascular & tumor	R	P, thalamus, internal capsule, lenticular nucleus	38 (97)	40.2	37 (38)	
Zoccolotti & Judica, 1991	SFES-P	Vascular	R	na	22 (26)	84.6	22 (22)	
Beschin & Robertson, 1997	Comb and Razor/Compact	Vascular	R	na	15 (31)	48.4	10 (15)	
McIntosh, Brodie, Beschin, & Robertson, 2000	Comb and Razor/Compact	Vascular	R	na	20 (31)	64.5	14 (20)	
Cocchini, Beschin, & Jehkonen, 2001	Fluff test; Comb and Razor/Compact	Vascular	R	na	12 (27)	44.4	10 (27)	
		Vascular	L	na	2 (11)	18.2	0 (2)	
Appelros et al., 2002	SFES-P, One item test	Vascular	R	na	18 (126)	14.3	18 (18)	
			L	na	4(146)	2.7	4 (4)	
Azouvi et al., 2002	One item test (eyes open)	na	R	na	na	16.0	na	
	One item test (eyes closed)					13.0		
Azouvi et al., 2003	CBS	Vascular	R	na	82 (83)	99.0	82 (82)	
Appelros et al., 2004	SFES-P	Vascular	R	na	23 (37)	62.5	23 (23)	
Beis et al., 2004	One item test (eyes open)	Vascular	L	F, P, T, O, internal capsule, thalamus	7 (78)	8.9	na	
	One item test (eyes closed)				10 (78)	12.8		
Buxbaum et al., 2004	Fluff test (modified)	Vascular	R	F, T, O, P, cingulate gyrus, periventricular white matter, basal ganglia, internal capsule, thalamus#	28 (166)	16.9	26 (28)	
Bowen, Gardener, Cross, Tyrrell, & Graham, 2005	One item test, Fluff test (modified), Face Washing	Vascular	R	na	20 (42)	47.6	12 (20)	
Azouvi et al., 2006	One item test (eyes open)	Vascular	R	F,T, subcortical	33 (206)	16.0	33 (33)	
			L		7 (78)	9.0	7 (7)	
	One item test (eyes closed)		R		27 (206)	13.0	27 (27)	
			L		10 (78)	12.8	10 (10)	
Glocker, Bittl, & Kerkhoff, 2006	CBS, Vest Test	Vascular	R	na	20 (25)	80	na	
			L		12 (25)	48		
	Fluff test (modified)		R		17 (25)	68		
			L		12 (25)	48		
Committeri et al., 2007	SFES-P	Vascular	R	P (postcentral and supramarginal gyrus), white matter supralenticular corona radiata, centrum semiovale##	30 (52)	57.7	22 (30)	
Lindell et al., 2007	SFES-P, One item test	Vascular	R	F, T, P, white matter	10 (34)	29.4	10 (10)	
Groh-Bordin et al., 2009	Vest Test	Vascular	L	F, white matter	7 (23)	30.4	na	
Baas et al., 2011	Fluff test	Vascular	R	T, P	7 (22)	31.8	7 (7)	
Fortis et al., 2010	CBS, One Item test (extended)	Vascular	R	F, P, T; white matter; basal ganglia	4 (10)	40	4 (4)	
Reinhart et al., 2012	Vest Test	Vascular	R	F, T, P, thalamus, basal ganglia	8 (8)	100	8 (8)	
Rousseaux, Sauer, Saj, Bernati, & Honoré, 2013	CBS	Vascular	R	F, T, P, rolandic cortex, centrum ovale, insula,internal capsule, striatum, thalamus	9 (15)	60.0	9 (9)	
	One Item test		R		1 (15)	7.0	1 (1)	
Caggiano, Beschin, & Cocchini, 2014	Comb and Razor/Compact	Vascular	R	F,T,P	27 (101)	26.7	na	
			L		21 (96)	21.9		
	Fluff test (eyes open)		R		23 (101)	22.8		
			L		21 (96)	21.9		
	Fluff test (eyes closed)		R		36 (101)	35.6		
			L		20 (96)	20.8		
Guariglia, Matano, & Piccardi, 2014	SFES-P	Vascular	R	F, T, P, O, insula, basal ganglia, internal capsula, lenticualr nucleus, corona radiata	122 (282)	43.3	95 (122)	
Palermo, Di Vita, Piccardi, Traballesi, & Guariglia, 2014	SFES-P, BRT	Vascular	R	F, T, P, O, internal and external capsula, corona radiata, basal ganglia, thalamus, insula	13 (32)	49.6	13 (13)	
Iosa, Guariglia, Matano, Paolucci, & Pizzamiglio, 2016	SFES-P	Vascular	R	na	35 (49)	71.4	35 (35)	
Rousseaux, Allart, Bernati, & Saj, 2015	One Item test, SSA, CBS	Vascular	R	F, P, T, temporo-occipital junction, white matter	29 (45)	64.4	21 (29)	
Besharati et al., 2016	One Item Test, Comb and Razor/Compact	Vascular	R	F (inferior / middle gyri), P (inferior), T (superior gyri), insula, dorsal-frontal white matter #	na (30)	na	na	
Moro et al., 2016	Comb and Razor/Compact	Vascular	R	F, P, T, superior temporal gyrus, insula, basal ganglia, corona radiata, external capsule	na (66)	na	na	
Spaccavento, Cellamare, Falcone, Loverre, & Nardulli, 2017	SFES-P	Vascular	R	na	60 (130)	46	45 (60)	
				Total number of patients	755 (2247)			

*SFES-P*, Semistructured Functional Evaluation Scale - Personal sub-scale; *BRT*, Body Representation Test; *SSA*, Subjective Straight Ahead; *CBS*, Catherine Bergego Scale; *F*, Frontal, *T*, temporal; *P*, Parietal; *O*, Occipital lobes; *R*, Right; *L*, Left; *PN*, Personal Neglect; *EN*, Extrapersonal Neglect; *na*, information not available

# sites of lesions of entire sample (i.e. not limited to patients showing PN)

## lesion sites for patients showing PN

i) ***Assessment methods***

Tables 1 and 2 report the assessment methods used in each study included in this review, and Table 3 provides a brief description of the most common tests used to assess personal neglect and how extensively they have been used.

**Table 3:** Description of assessment methods for personal neglect

Author/s	Method	Description	N. of studies^#^
Bisiach et al., 1986a, b	One item test	Patients are asked to reach with their ipsilesional hand toward the contralesional hand. A four level scale is used, ranging from 0 (normal performance) to 3 (no attempt to reach the target hand).	23
Zoccolotti & Judica, 1991	Semistructured Functional Evaluation Scale - Personal subscale	Three objects are presented once at a time: a comb, a razor (for male)/powder (for female) and a pair of eyeglasses. Patients are asked to demonstrate to the experimenter how to use them. The performance is scored from 0 (normal performance) to 3 (severe deficit).	14
Beschin & Robertson, 1997; McIntosh et al., 2000	Comb and Razor/Compact test	Two objects are presented once at a time: a comb and a razor (for male)/facial compact (for female). Patients are asked to use the objects for a fixed time. The proportion of strokes on each side (for both comb and razor/compact) is counted.	12
Cocchini et al., 2001	Fluff test	Blindfolded patients are asked to remove, with their ipsilesional arm, 24 cardboard stickers attached to the front of their clothes. There are 15 targets on the left side of the body (3 on the central body midline area, 6 on the arm and 6 on the leg) and 9 on the right side (no targets are placed on the right arm). A score lower than 13 on the left-hand side of the body is considered pathological.	11*
Fortis et al., 2010; Sambo et al., 2012	One item test (extended version)	Patients are asked to reach with their ipsilesional hand towards six contralesional body parts (ear, shoulder, elbow, wrist, waist, knee). A four level scale is used, each response is scored as follows: 0 (no attempt to reach the target), 1 (search without reaching), 2 (reaching with hesitation and search) and 3 (normal performance), with a 0 to 18 score range.	2
Daurat-Hmeljiak, Stambak, & Berges, 1978; Guariglia & Antonucci, 1992	Body Representation test	Two different tasks are performed: naming-localisation of the tiles and construction of the frontal and profile view of the human body and head.	5
	Frontal body-evocation	A wooden board with a head depicted on it and nine tiles, each representing a part of the human body (left and right legs, hands, arms, parts of the chest and the neck) are presented to the patients. One tile at a time is shown to patients, whom are asked to name the body part before putting it on the board. The position of the tile is recorded and the previous tile is removed. The score is the sum of the correctly placed tiles (maximum score: 9).	
	Lateral body-evocation (left and right)	For the lateral body-evocation test, the subject has to choose among different views of the same body part before putting the tile on the table (for example, the arm is presented in frontal, lateral right, and lateral left views) (maximum score: 4).	
	Frontal face-evocation	The contour of the face and 12 tiles, each representing a part of the face (nose, mouth, chin, eye browns, hair) are presented. The procedure is the same as for the frontal body-evocation test (maximum score: 12).	
	Lateral face-evocation (left and right)	For the lateral face-evocation test, the subject has to choose among different views of the same body part before putting the tile on the table (for example, the nose is presented in frontal, lateral right, and lateral left views) (maximum score: 6).	
Bergego et al., 1995	Catherine Bergego Scale	Direct observation (and rating) of the patient’s functioning in 10 real-life situations such as grooming, dressing, or wheelchair driving. The same questionnaire is administered to patients (self-evaluation) and carers.	5
Goodenough, 1926; Chen-Sea, 2000	Draw-A-Man test	Using a blank piece of paper and a pencil, patients are asked to draw an entire man figure. The total score is 10, one point is given to each of the following body parts: head, trunk, right arm, left arm, right hand, left hand, right leg, left leg, right foot, and left foot.	1
Buxbaum et al., 2004	Fluff test (modified version)	Blindfolded patients are asked to remove 6 cotton balls placed on the left side at the shoulder, chest, elbow, forearm, wrist and hip.	1
Bowen et al., 2005	Fluff test (modified version)	Blindfolded patients are asked to remove 12 stickers placed only on the upper half of the body, in order to avoid the need for participants to get out of bed.	1
Bowen et al., 2005	Face Washing	Patients are asked to wipe their face for 20 s using a sponge held in the right hand. The performance is recorded. Any bias in the area covered and the proportion of time spent on the left is scored.	1
Glocker et al., 2006	Fluff test (modified version)	The 24 targets are attached to a jacket and the front side of a trouser before examination and not on the patient’s body surface (as is done in the standard Fluff test).	1
Glocker et al., 2006	Vest test	Patients are asked to search and hand to the experimenter as quickly as possible, with their ipsilesional arm, 24 objects (12 on either side of the trunk) placed in pockets on the front side of a vest.	1
Richard et al., 2004	Subjective Straight-Ahead	Patients are asked to imagine a virtual line starting from their umbilicus and extending away straight ahead of the trunk. In a dark room they have to adjust, with the right hand, the position of a luminous rod which can be rotated and translated along a plate in such a way that its two extremities stood on the virtual line.	1

Test are shown according to frequency across studies (most frequent at the top and less frequent at the bottom). Tests with the same frequency have been arranged chronologically

*In some studies patients performed the Fluff test with open eyes

# Number of studies where the test has been used. Note that some studies used more than one task

Despite a range of diagnostic methods having been developed to assess personal neglect and distortion of personal domain (see Table 3), in the majority (57%; 29/51) of the single case studies reported in Table 1, personal neglect was diagnosed by means of clinical observation. Only 20% (10/51) of the studies used one psychometric test, and as few as 22% (11/51) of the studies used two or three tests to assess personal neglect (seven and four studies respectively). In the group studies (Table 2) personal neglect was assessed more systematically with nearly all studies adopting at least one psychometric test (97%; 29/30). In about a third (30%; 9/30) of the studies personal neglect was investigated by means of two different tests, and only 7% (2/30) of the studies used three different measures to assess personal neglect. This is rather surprising if we consider that other forms of neglect, such as extrapersonal neglect, are usually examined through the use of a wider battery of tests to reflect the complexity of extrapersonal neglect (e.g., Behavioural Inattention Test; Wilson, Cockburn, & Halligan, 1987). Interestingly, when more than one personal neglect assessment method was used, the researchers observed a heterogeneous picture (e.g. Bowen et al., 2005; Azouvi et al., 2006; Glocker et al., 2006; Rousseaux et al., 2013; Caggiano et al., 2014), suggesting that personal neglect may be a term that encompasses disruption of different underlying mechanisms.

A possible reason for the lack of systematic investigations into the literature concerning personal neglect may be due to the fact that personal neglect was not investigated in group studies until 1978 (Cutting, 1978), more than 60 years after its first description. During this period, personal neglect was mainly described in single case studies where the research focused on the clinical evidence for this syndrome (Table 1). One of the first attempts to systematically investigate personal neglect was not until 1986 when Bisiach and colleagues (Bisiach et al., 1986a) devised the *One Item Test* (Table 3) and reported that 39% of 97 patients showed personal neglect. The *One Item test* represents a very easy and quick way to assess evidence of personal neglect and has been extensively used for clinical and research purposes in at least 23 studies. This scale has been recently modified to allow evaluation of other body parts such as ear, shoulder, elbow, wrist, waist and knee (Fortis et al., 2010). However, the test also presents some limitations. It usually consists of a single trial assessing a limited area, such as the contralateral arm. More importantly, the command per se (i.e. “With this hand, touch your other hand”) implies the existence of the contralesional limb. This may result in the patient attempting to reach for a limb, otherwise neglected, thus potentially leading to a false negative result. Finally, the search for the contralesional limb is guided by proprioceptive information providing little insight into the patient’s mental representation of their own body (Cocchini et al., 2001).

To respond to some of these limitations, Zoccolotti and Judica (1991) devised the *Semistructured Functional Evaluation Scale* (SFES), which includes a semi-structured scale assessing difficulties relating to personal space*.* The ‘Personal sub-scale’ (SFES-P) requires the use of everyday objects, such as a comb, a razor (for males), powder (for women) and glasses, with the examiner assigning a score from 0 (normal performance) to 3 (severe personal neglect). The rating, depending on the examiner’s expertise, provides a general indication of the presence or absence of personal neglect but this rating seems less able to establish different degrees of personal neglect severity, resulting in an underestimation of less severe forms (Beschin & Robertson, 1997).

To improve the objectivity of the assessment of personal neglect, Beschin and Robertson (1997) developed the *Comb and Razor/Compact Test* where the examiner considers the number of strokes applied by the patient on each side of their own face (when asked to pretend to shave or apply make-up) or on each side of their head (when asked to pretend to comb their hair). Patient’s performance was then compared with normative data and 48% of patients with right-sided brain damage had a pathological performance in the Comb and Razor/Compact Test. Interestingly, in the same study 12 patients showed a double dissociation between extrapersonal neglect and personal neglect. The scoring was later revised by McIntosh and colleagues (McIntosh et al., 2000), who proposed a new formula and cut-off criterion to account for possible biases due to ambiguous response strokes (i.e. responses not clearly assigned on either side).

It must be considered though, that both the Comb and Razor/Compact test and the SFES-P did not extend the investigation to other parts of the body. Such investigation may be crucial as it is widely accepted that personal neglect also refers to lack of exploration of part of the body. To this aim, Cocchini and colleagues (Cocchini et al., 2001) developed the *Fluff Test*, where blindfolded patients were required to remove 24 circles previously attached on the patient’s clothes in order to cover the contralesional arm, the torso and both legs. Scoring was based on the number of targets removed on each side and the diagnostic cut-off was based on normative group performance. Clinical findings on 38 brain-damaged patients (27 with right and 11 with left unilateral lesions) confirmed a relatively high percentage of personal neglect (44%) following right brain lesion and a lower, though not negligible, percentage (20%) following left-sided brain damage. In Cocchini and colleagues (Cocchini et al., 2001) study the authors also reported six cases of double dissociation between extrapersonal neglect and personal neglect, and a low correlation between these two forms of neglect. These findings seem consistent with previous studies also showing dissociations between the personal and extrapersonal spaces (Guariglia & Antonucci, 1992; Peru & Pinna, 1997; Bisiach et al., 1986a; Beschin & Robertson, 1997; Bailey, Riddoch, & Crome, 2000). However, Glocker and collaborators (Glocker et al., 2006) reported a considerably higher frequency of personal neglect assessed by means of the *Vest Test,* where patients are asked to search for 24 everyday objects hidden in 24 pockets on a vest, and a modified version of the Fluff test, where targets were attached on a long jacket worn by the patients. In a sample of 25 patients with right-sided brain damage and 25 with left-sided brain damage, up to 80% of the patients with right-sided damage and 48% of those with left-sided damage showed personal neglect on the Vest test, whereas up to 68% of the patients with right-sided damage and 48% of those with left damage showed personal neglect when the modified Fluff test was used. The different findings on frequency may be due to different inclusion criteria used in the studies. However, it should be a matter of careful consideration if relatively small methodological changes, as those between the Vest test and the modified Fluff test, have led to such different diagnostic outcomes in the same sample (e.g. 80% versus 68% of those with right damage). Contradictory results have been observed also in a study by Bowen and collaborators (Bowen et al., 2005) when personal neglect was assessed by means of the *Face Washing test,* where patients are asked to wipe their face for 20 s with the right hand. In this test, the time spent on the left side of the face is compared with the time spent on the right side. Results of the pilot study did not seem to be in line with other personal neglect tests adopted for the preliminary assessment, such as the *One Item Test* and the *Fluff test*. The authors considered the duration of the test as a possible methodological bias. Indeed, 20 s may be an unusually long time to wipe one’s own face and this may have led the patients to clean their face more carefully than they would normally do, spending more time and eventually moving on to the left areas. Unfortunately, the authors only reported data on the total time spent on either the left or right side of the face, not providing information on potential differences between each side of the face during the first few seconds of the task. Focusing on a shorter window of time could have provided valid data about latent personal neglect and a modified version of ths task may represent a further valuable measure to assess personal neglect.

In line with the attempt to maintain some type of ecological validity in assessing personal neglect, methods requiring meta-cognition evaluation, such as the Catherine Bergego Scale (Bergego et al., 1995; Azouvi et al., 2003) have been introduced. The Catherine Bergego Scale is a 10-item questionnaire which takes into account several dimensions of neglect within daily living: personal and motor neglect, extrapersonal, peripersonal and anosognosia. Therapists and patients are asked to rate possible difficulty on a series of situations, such as “Forgets to clean the left side of his or her mouth after eating”. The authors stressed the importance of this particular questionnaire in comparison to conventional tests, which, they argued, fail to consider a patients actual performance in their everyday activities, yet real-life observations may pick up neglect in a way that is missed in “simulated tasks” (Azouvi, 2016).

All the tests described above require actions toward the contralesional body, or an evaluation of these type of actions, but a very different approach to assess possible distortion of personal domain, has been focusing more on the pictorial component of prototypical body representation. At the end of the 1970s, it was proposed that neglect could involve a deficit of internally generated images, namely, the representational hypothesis (Bisiach & Luzzatti, 1978; Bisiach, Luzzatti, & Perani, 1979, Bisiach, Capitani, Luzzatti, & Perani, 1981). Therefore, the impairment may involve a distortion of mental events the occurrence of which is not necessarily linked to the actual stimulation or actions (Bisiach & Luzzatti, 1978). In line with this well-documented approach to neglect, some personal neglect tests (Table 2) have been developed to investigate the mental representation of one’s own body.

These studies raise an interesting question about the extent of personal neglect and its implications for mental representations of one’s own body and represent a valuable source of information for research purposes. However, the relevance of these tasks to diagnose personal neglect is debatable as these methods do not distinguish possible confounding variables related to forms of neglect in other domains (e.g. peripersonal space and arm-reaching space). In this vein, some tests have been developed to investigate body representation and been used as diagnostic tools (Table 3). For example, the *Draw-A-Man* test (Goodenough, 1926) represents one of the most controversial methods as it has been often used for neuropsychological evaluation in patients with brain-damaged (Andrews, Brocklehurst, Richards, & Laycock, 1980; Cohn, 1953; Colombo, De Renzi, & Faglioni, 1976; Gasparrini, Shealy, & Walters, 1980; Reznikoff & Tomblen, 1956; Riklan, Zahn, & Diller, 1962; Schulman, Kaspar, & Throne, 1965; Ska & Nespoulous, 1987). However, the Draw-A-Man test only recently has been considered as a diagnostic method for personal neglect. Chen-Sea (2000) proposed to validate this test by assessing the presence of personal neglect in right-sided brain damage patients. Patients were asked to draw a human figure on a blank piece of paper and any asymmetry, independently evaluated by two raters, was considered evidence of personal neglect. The results of the study showed the presence of personal neglect in 13 patients out of 51 (25.5%). However, it should be highlighted that the possible bias due to associated representational neglect and extrapersonal neglect remains unclear, and it is difficult to exclude any role of these latter forms of neglect on the test findings. In addition to this, results of the Draw-A-Man test have not been compared with other tests for personal neglect, leaving its specificity unclear. Another interesting assessment tool for personal neglect is the *Body Representation* test (Daurat-Hmeljiak et al., 1978), which consists of four subtests requiring the patient to name, localize and reconstruct specific body parts. In order to find the correct position of a specific body part, patients with personal neglect cannot consider the relations among body parts separately from their own body (allocentric frame of reference), but they have to use an egocentric reference (their own body). Therefore, Palermo et al. (2014) claimed that this test necessitates the use of an egocentric frame of reference in processing the topological body map. This test has been used in several studies with brain-damaged patients (Canzano, Piccardi, Bureca, & Guariglia, 2011; Guariglia & Antonucci, 1992; Marangolo et al., 2003; Palermo et al., 2014) but validation for clinical assessment of this test has not been undertaken as yet.

Similarly, the *Subjective Straight Ahead* test evaluates the egocentric frame of reference (Heilman, Bowers, & Watson, 1983; Karnath, Sievering, & Fetter, 1994; Richard, Honoré, & Rousseaux, 2000; Richard, Rousseaux, Saj, & Honoré, 2004; Rousseaux et al., 2015). In this task, patients with neglect tend to show a bias towards the right when asked to point ‘straight ahead’ of their body midline. This bias seems to be closely linked to an alteration of the internal representation of the midsagittal plane of the body, which is the egocentric frame of reference. Richard et al. (2004) devised a test to obtain a direct indication of the Subjective Straight Ahead test, in which participants were asked to adjust the position of a luminous rod straight ahead of the middle part of their trunk in a dark room. Results showed a translation of the egocentric frame of reference towards the right. In a study conducted by Rousseaux et al. (2015), the same Subjective Straight Ahead test procedure developed by Richard and colleagues was used to assess personal neglect together with the One Item Test (Bisiach et al., 1986a). In a sample of 45 patients with right-sided brain damage, the authors reported eight patients as having personal neglect (18%) based on their impaired performance in the One Item Test and that up to 27 (60%) of these patients showed a pathological rightward deviation in the Subjective Straight Ahead test. It must be considered that the tasks assessing distortion of body representation and egocentric reference frames inevitably require a crucial involvement of information arising from extrapersonal space and they tend to address different aspects than premotor personal neglect. Overall, these measures therefore provide valuable research information about some aspects of personal space, but their specificity in assessing personal neglect remains to be clarified.

ii) ***Neural correlates of Personal Neglect***

As discussed above, without a robust diagnostic method for personal neglect, it is difficult to evaluate the neural correlates of personal neglect as, in some studies, its diagnosis is not well defined, leaving unresolved doubts of false negatives or false positives. A further reason for caution is the fact that several studies report limited information on the patient lesions, or the neuroimaging investigation performed is represented by a routine radiological exam. Keeping in mind these limitations, we have reviewed the literature to consider convergence of outcomes and replication of findings.

Considering Table 1, the vast majority of the single and multiple cases (74/83; 89%) suffered from unilateral right-sided brain damage, whereas only 11% of the sample showed personal neglect associated with bilateral (four cases) or unilateral left-sided brain damage (five cases). Similarly, in Tables 2, 72% (1624/2247 patients) of the patients in the 30 group studies reported personal neglect following right-sided brain damage, whereas only 20% (457/2247) following left-sided damage. Therefore, while there is a clear predominance of personal neglect following right-sided brain damage, about one-fifth of patients showed evidence of this form of neglect after left-sided lesions. Traditionally, personal neglect is investigated following right-sided brain damage and assessed following left-sided injury only if the authors have specific research questions or, when it is evident on general assessment (i.e. severe). On the basis of such consideration, we suspect that personal neglect after left-sided brain injury may be more frequent than reported.

To further explore the hemispheric asymmetry of personal neglect, we considered five studies reported in Table 2 where personal neglect was investigated by means of the same method both in patients with left-sided or right-sided brain damage (Cocchini et al., 2001; Appelros et al., 2002; Azouvi et al., 2006; Glocker et al., 2006; Caggiano et al., 2014). These five studies investigated personal neglect in a total sample of 841 patients, of which 485 had right unilateral lesions and 356 had left unilateral lesions. When different tests were used to assess personal neglect in the same sample, resulting in slightly different frequencies of personal neglect, we considered the highest value reported from each sample. Evidence of personal neglect was found in 119 out of 485 patients with right-sided brain damage (24.5%) and 49 out of 356 (14%) patients with left-sided lesions. Personal neglect after right hemisphere lesions was significantly more frequent than after left hemisphere lesions (χ^2^ = 13.17; *p* <. 001). However, the frequency of personal neglect in these samples should be interpreted with caution as they are not epidemiological studies, therefore inclusion criteria for right-sided damage vesus left-sided damage may differ. Unintended sampling bias could account, at least in part, for the higher frequency of personal neglect in one group. Interestingly, though personal neglect seems to be more often associated to right-sided brain damage, the presence of personal neglect is highly variable ranging from 2.7% (Appelros et al., 2002) to 48% (Glocker et al., 2006). A recent study by Kesayan and collaborators (Kesayan, Lamb, Williamson, Falchook, & Heilman, 2016) investigated personal pseudoneglect in 24 healthy participants who showed the typical leftward deviation on allocentric neglect tasks, such as line bisection. However, when they were asked to perform egocentric neglect tasks, such as evaluating the strength of tactile stimuli and the arm bisection test, the participants showed a rightward deviation. The authors concluded that the right hemisphere is crucial for modulating allocentric attention, whereas the left hemisphere may be more involved in tasks requiring egocentric attention and attention directed to personal space (Kesayan et al., 2016).

Information about site of lesions within the hemispheres is even less conclusive and it is complicated by the lack of clear information about the lesion site in some studies, as well as the association of other deficits, in particular extrapersonal neglect, making it difficult to understand the selective impact of a lesion on personal neglect. An analysis of single cases, which tend to provide more detailed descriptions, was also rather inconclusive. Considering the single case studies reported in Table 1, the lesion site information was not available for 10 cases (Zingerle, 1913; Kramer, 1915; Barré et al., 1923; Barkman, 1925; Ehrenwald, 1930; Ehrenwald, 1931; Von Hagen & Ives, 1937; Gerstmann, 1942). Three further studies provide limited lesion site information (Paulig et al., 2000; Di Vita et al., 2016; Ronchi et al., 2017) and in one study localization of the brain lesion was deduced through clinical diagnosis (Wortis & Dattner, 1942). Based on the information reported in the other 65 cases, personal neglect seems frequently associated with a widespread range of cortical and subcortical lesion sites, including the thalamus, basal ganglia and internal capsule.

A similar picture appears when we considered group studies reported in Table 2. Lesion site information is not available in 12 studies (Cutting, 1978; Zoccolotti & Judica, 1991, Beschin & Robertson, 1997; McIntosh et al., 2000; Cocchini et al., 2001; Appelros et al., 2002, 2004; Azouvi et al., 2003; Bartolomeo, Sylvie Chokron, & Gainotti, 2001; Bowen et al., 2005; Glocker et al., 2006; Spaccavento et al., 2017) and various papers reported widespread lesions across the four lobes and subcortical structures, including the thalamus, basal ganglia, insula and white matter fibers. However, a few recent group studies start to shed light on the identification of different neural correlates between personal and extrapersonal neglect. Baas et al. (2011), using subtractive analysis to highlight the affected areas in patients with personal neglect, observed that the temporo-parietal junction and the underlying white matter were most affected (about 50% more often) in patients with personal neglect compared to patients without personal neglect. One of the first systematic studies on neural correlates of personal neglect in humans was conducted by Committeri and colleagues (Committeri et al., 2007). The authors observed a significant dissociation between the neural substrates underlying personal versus extrapersonal neglect. In particular, the authors reported that while lesions involving frontal circuits such as the right central premotor cortex, medial frontal gyrus and superior temporal regions are related to extrapersonal neglect, the critical regions related to personal neglect seem to be the postcentral and supramarginal gyri of the parietal lobe and white matter that underlies the two regions. Interestingly, damage to the right inferior posterior-parietal region (supramarginal gyrus), and underlying white matter, has been considered a pathological correlate of asomatognosia (Feinberg, Roane, & Ali, 2000; Feinberg, DeLuca, Giacino, Roane, & Solms, 2005).

The supramarginal gyrus has a considerable hemispheric specialization, the left side is associated with linguistic information processing, while right side is more related to spatial information processing (Kandel, Schwarts, Jessel, Siegelbaum, & Hudspeth, 2012). In detail, the main afferent signals to the supramarginal gyrus come from the somatosensory cortex (post-central gyrus) concerning the positions of the limbs. In addition, it receives information from the vestibular system and the premotor areas about head orientation in space. The supramarginal gyrus sends information to both the associative somatosensory cortex, involved with visual information processing concerning the spatial location, and to the premotor areas, involved in spatial orientation and movement planning. The postcentral gyrus, receives sensory and proprioceptive information from the body surface and tension state of joints. In addition to this, efferent fibers projecting to the primary motor cortex allow the motor system to integrate information about the intention of movement with proprioceptive information. As originally proposed by Committeri and colleagues (Committeri et al., 2007), these observations support the hypothesis that personal neglect is due to a functional disconnection between regions important for coding proprioceptive and somatosensory input, such as the postcentral gyrus, and those which encode more abstract egocentric representation of the body in space, such as the supramarginal gyrus (Coslett, 1998; Galati, Committeri, Sanes, & Pizzamiglio, 2001; Committeri et al., 2018). This type of injury could cause an impairment in creating a mental model of body image, awareness of the configuration and motion of body in space (Galati et al., 2001).

Similar anatomical substrates, with no apparent involvement of the supramarginal gyrus, have been recently reported in a study conducted by Rousseaux et al. (2015). In a sample of 45 neglect patients, Rousseaux and colleagues observed a dissociation between the neural correlates underlying personal neglect and peripersonal neglect. In particular, personal neglect was associated mainly with lesions in somatosensory cortex and motor cortex and, to a lesser degree, in superior temporal gyrus, middle temporal gyrus, intraparietal sulcus and inferior parietal gyrus. On the other hand, peripersonal neglect was associated with much larger lesions involving the superior temporal gyrus and inferior parietal gyrus with extension to the middle temporal gyrus, temporo-occipital junction, somatosensory and motor cortices.

Despite some methodological limitations concerning the assessment of personal neglect (see Assessment methods, above), these data stress the relevance of parietal lesions in personal neglect and partially support the hypothesis that body centered tasks, which require conscious awareness of body representation and an egocentric coding of spatial information, are affected by lesions involving the anterior parietal lobe, superior temporal lobe and the underlying white matter (Galati et al., 2001).

iii) ***Personal and extrapersonal neglect***

Unilateral neglect may affect both personal and extrapersonal spaces and they have often been associated (e.g. Bisiach et al., 1986a; Cocchini et al., 2001; Committeri et al., 2007; Guariglia et al., 2014; Iosa et al., 2016). Unsurprisingly, information provided in Table 1 seems to confirm that personal and extrapersonal neglect frequently co-occur. Indeed, these two forms of neglect were both assessed in 74 cases (Table 1), and up to 63 patients (85%; 63/74) showed personal neglect associated with extrapersonal neglect. Despite this, evidence of selective personal neglect was not infrequent (Bisiach et al., 1986a; Buxbaum et al., 2004). We identified evidence of selective personal neglect in 11 single cases (Table 1; Garcin et al., 1938; Zoccolotti & Judica, 1991; Guariglia & Antonucci, 1992; Beschin et al., 1997; Peru & Pinna, 1997; Marangolo et al., 2003; Ortigue et al., 2006; and four patients in Di Vita et al., 2016) and 10 group studies (see Table 2; Bisiach et al., 1986a; Beschin & Robertson, 1997; McIntosh et al., 2000; Cocchini et al., 2001; Buxbaum et al., 2004; Bowen et al., 2005; Committeri et al., 2007; Guariglia et al., 2014; Rousseaux et al., 2015; Spaccavento et al., 2017). We fully agree with Guariglia and Antonucci (1992) who suggested that the number of patients showing a pure form of personal neglect may be underestimated due to methodological issues, as there is a lack of measures for evaluating impaired processes related to the contralesional body space and, as discussed in the Assessment of personal neglect section, above, the evaluation of personal neglect is still associated with some important limitations. Moreover, there is a small, but growing, body of evidence reporting a pattern of double dissociation between personal versus extrapersonal neglect (mainly peripersonal: Patterson & Zangwill, 1944; Rizzolatti, Matelli, & Pavesi, 1983; Bisiach, Perani, et al., 1986; Zoccolotti & Judica, 1991; Guariglia & Antonucci, 1992; Pizzamiglio et al., 1989; Vallar, Sterzi, Bottini, Cappa, & Rusconi, 1990; Beschin & Robertson, 1997; Cocchini et al., 2001; McIntosh et al., 2000; Bowen et al., 2005; Committeri et al., 2007; Spaccavento et al., 2017). Pizzamiglio and colleagues (Pizzamiglio, Guariglia, Antonucci, & Zoccolotti, 2006) reviewed the effect of rehabilitation methods, such as transcutaneous electrical nervous stimulation and optokinetic stimulation, on different aspects of neglect syndrome. The authors concluded that some of these techniques might lead to a significant recovery of spatial exploration for the extrapersonal space but have little, if any, impact on representational neglect and personal neglect. This research suggests that different underlying mechanisms may underpin these forms of neglect. More recently, Iosa et al. (2016) investigated the recovery of personal and extrapersonal neglect in 49 patients with right-sided brain damage following a combination of physiotherapy and a 6-week neuropsychological rehabilitation training, which involved visual scanning, reading, copying verbal and non-verbal material and verbal description of scenes. On average the extrapersonal neglect improvement was almost 80%, whereas personal neglect recovery was about 58% in patients who initially showed mild personal neglect and up to 72% in those who initially showed severe personal neglect. However, the authors found no correlation between personal and extrapersonal neglect improvement, supporting once more the hypothesis for different mechanisms, at least for recovery.

Therefore, the frequent association of personal with extrapersonal neglect seems to indicate a shared attentional deficit which can involve different sectors of the space (Vallar & Bolognini, 2014). However, there is also growing evidence suggesting that, in addition to the inattentional component, personal neglect might also subtend a disorder of multisensory body representation. Coslett, Saffran, and Schwoebel (2002) suggested that personal neglect patients might show a disorder of body representation mainly due to impaired sensorimotor information, that is, impaired perception of body schema. This consideration was based on a previous study where patients were asked to decide whether displayed images of hands (on dorsal and palmar view) were left or right hands (Coslett, 1989). This type of task required participants to form a mental representation of this part of the body in order to make a correct judgment (Parsons, 1987a; b). This result does not seem to be attributed to a loss of information about the representation of the left side of the body but to an impairment in representing the correct relations amongst different parts of the body (Rousseaux et al., 2013). Interestingly, passive limb activation in personal neglect patients, but no general alertness cueing, reduced judgment error rates in discriminating left hand stimuli, suggesting that “bottom-up” interventions in brain-damaged patients displaying personal neglect, modulates the activation of the body schema and can promote a better body representation (Reinhart et al., 2012).

Guariglia and Antonucci (1992) described a patient showing a profound distortion of the left side of his body representation in absence of extrapersonal neglect. The authors argued that personal neglect might be linked to an alteration of bodily spatial relations and other authors (Baas et al., 2011) supported this interpretation, further claiming that body representation is a fundamental mechanism in personal neglect, which could be interpreted as a disorder of the ‘body schema’ and implying that this construct involves mechanisms that go beyond the mere higher order somatosensory representation (Coslett et al., 2002).

## Conclusions

Personal neglect represents an important aspect of everyday life (e.g., dressing, washing, eating, and personal care) and can be detrimental to functional rehabilitation (Iosa et al., 2016). However, in the last 123 years, the personal neglect has been relatively ‘neglected’ in research and clinical practice. The number of studies that have systematically investigated this syndrome is vastly smaller compared to those investigating the extrapersonal neglect. The variety of diagnostic measures to assess personal neglect is also limited, with only 7% of the group studies adopting more than two assessment tools. Although some recent studies have adopted a more comprehensive approach, the need for novel and sensitive diagnostic tools to assess various aspects of personal neglect has been emphasized by various authors and echoed in this review. A greater attention to the assessment issue seems to represent the first and crucial step to refine methodological aspects of research on personal neglect. Different aspects of extrapersonal space have been extensively studied and examined by means of standardised test batteries, which have been widely used for decades for research and diagnostic purposes. On the contrary, the lack of uniformity in the assessment methods concerning personal neglect has led to unclear definitions of such impairment, undermining its relevance for clinical and research purposes. A robust assessment method, with batteries of tests evaluating different aspects and body areas will enhance our ability to diagnose different degrees of personal neglect and consequently, it will lead to a better definition of related neuronal substrates and networks. As suggested by Committeri and colleagues (Committeri et al. 2018), the use of the One Item Test (Bisiach et al., 1986a, b) and the SSES-P (Zoccolotti & Judica, 1991) or the Comb and Razor/Compact test (Beschin & Robertson, 1997; McIntosh et al., 2000) should be part of the standard assessment of personal neglect. However, as highlighted in the present review, personal neglect refers to lack of exploration of part of the body. Therefore, we believe that the Fluff test (Cocchini et al., 2001) should not be just desirable (Committeri et al., 2018) but an integral part of the routine assessment, in order to have a triad of tests that target the different body areas providing a more exact clinical picture of personal neglect.

This review also shows that, despite a frequent co-occurrence of personal and extrapersonal neglect, a few cases show a selective form of personal neglect suggesting that these two forms of neglect can be dissociated. Also, the neural bases of personal and extrapersonal space awareness seem to support the hypothesis that these two forms of neglect may, at some point, underlie different neuroanatomical structures and processes. Similarly, based on the body representation literature, it seems that personal neglect may not differ from extrapersonal neglect only because it represents an attentional deficit involving a specific space frame (i.e. the body). On the contrary, the impairment associated with personal neglect may go beyond an attentional deficit and be part of a more complex deficit of body representation (Committeri et al., 2018). The impairment of personal space processing is indeed associated with a perturbation of the mental representation of the patient’s body. In fact, personal space awareness requires the integration of many sources of proprioceptive and somatosensory information pertaining to the body as well as abstract egocentric representations necessary to perceive and to move the body in space (Committeri et al., 2007). Impairments in use of this complex array of information may lead to a significant reduction of awareness for possible impairment related to the neglected side of the body.

In more dramatic cases, the loss of awareness of one body-half can be associated with profoundly disturbed feelings of ownership of contralesional limbs (Dieguez, Staub, & Bogousslavsky, 2007; Pinel, 2009). Patients do not recognise that some parts of their (usually left) body belong to them, as they show asomatognosia (Jenkinson, Moro, & Fotopoulou, in press; Arzy, Overney, Landis, & Blanke, 2006). The relationship between personal neglect and asomatognosia is still controversial, as some studies have described a defective sense of ownership in the absence of a disturbance in body awareness (Hécaen, Ajuriaguerra, Guillant, & Angelergues, 1954; Bisiach, Rusconi, & Vallar, 1991; Halligan, Marshall, & Wade, 1993; Bisiach et al., 1990).

A better understanding of personal neglect will also shed light on syndromes often associated, and sometimes confused, with personal neglect, such as asomatognosia, anosognosia and other syndromes showing disturbances of personal identity.
